# The melanin-concentrating hormone system as a target for the treatment of sleep disorders

**DOI:** 10.3389/fnins.2022.952275

**Published:** 2022-09-13

**Authors:** Liam E. Potter, Christian R. Burgess

**Affiliations:** ^1^Department of Molecular and Integrative Physiology, Michigan Medicine, Ann Arbor, MI, United States; ^2^Michigan Neuroscience Institute, University of Michigan, Ann Arbor, MI, United States

**Keywords:** melanin-concentrating hormone, MCH, MCHR1, sleep, sleep disorders, pharmacotherapy, REM, drug development

## Abstract

Given the widespread prevalence of sleep disorders and their impacts on health, it is critical that researchers continue to identify and evaluate novel avenues of treatment. Recently the melanin-concentrating hormone (MCH) system has attracted commercial and scientific interest as a potential target of pharmacotherapy for sleep disorders. This interest emerges from basic scientific research demonstrating a role for MCH in regulating sleep, and particularly REM sleep. In addition to this role in sleep regulation, the MCH system and the MCH receptor 1 (MCHR1) have been implicated in a wide variety of other physiological functions and behaviors, including feeding/metabolism, reward, anxiety, depression, and learning. The basic research literature on sleep and the MCH system, and the history of MCH drug development, provide cause for both skepticism and cautious optimism about the prospects of MCH-targeting drugs in sleep disorders. Extensive efforts have focused on developing MCHR1 antagonists for use in obesity, however, few of these drugs have advanced to clinical trials, and none have gained regulatory approval. Additional basic research will be needed to fully characterize the MCH system’s role in sleep regulation, for example, to fully differentiate between MCH-neuron and peptide/receptor-mediated functions. Additionally, a number of issues relating to drug design will continue to pose a practical challenge for novel pharmacotherapies targeting the MCH system.

## Introduction

Insufficient or disordered sleep is extremely common and consequential to those affected, and to society at-large. The scope of the problem is immense—the United States Centers for Disease Control and Prevention (CDC) recently reported that 1 in 3 Americans is unable to obtain sufficient sleep on a regular basis. Those who fail to get sleep of sufficient duration or quality will likely be afflicted by significant impairments in their daytime functioning, which can lead to accidents, poor performance and decision making, and other negative consequences. The aggregate economic burden associated with sleep disruption is estimated at one hundred billion dollars per year in North America (Wickwire et al., 2016). In addition to affecting daytime performance, lack of sleep can impact other aspects of health, worsening existing conditions and predisposing individuals to a variety of physiological or neurological issues (e.g., cardiovascular problems, depression and anxiety). A variety of common sleep disorders cause excessive daytime sleepiness and other attendant symptoms. Insomnia is generally considered the most common sleep-disorder, with roughly 30–60% of the population reporting symptoms at some point each year, and between 10 and 33% prevalence on a given day (Bhaskar et al., 2016; Dopheide, 2020). Of similarly high incidence and prevalence is obstructive sleep apnea (OSA), in which breathing is disrupted or interrupted during sleep, leading to fragmented sleep. Recent studies have estimated that up to 30–50% of the population suffer from OSA, and up to 10–20% from the more severe OSA syndrome (Franklin and Lindberg, 2015). Between 4 and 15% of the population suffer from restless legs syndrome (RLS)—a sleep-related disorder characterized by periods of intense discomfort in the legs, along with an urgent need to move them (Ohayon et al., 2012). As with insomnia, both OSA/RLS can cause sleep loss, leading to excessive daytime sleepiness and other associated problems. In addition, there are various rapid eye-movement (REM) and non-REM sleep-associated parasomnias which are relatively common (0.5–15% prevalence)—including sleep-paralysis, sleepwalking, night terrors, and REM sleep behavior disorder. Narcolepsy, a chronic sleep disorder characterized by excessive daytime sleepiness, is rarer, occurring in roughly 1 in 2000 individuals (0.05%). Whether widespread or relatively rare, all of these disorders can be profoundly disruptive to the lives of those afflicted. The debilitating nature of these disorders, and the inadequacy of most currently available treatments, make it imperative for the research community to seek out and evaluate novel therapeutic avenues. Fortunately, basic research into the neurobiology of sleep, and sleep disorders, continues to reveal potential pharmacological targets. Among the various targets currently being evaluated for their utility in the treatment of sleep disorders, the melanin-concentrating hormone (MCH) system holds considerable promise.

Melanin-concentrating hormone (MCH) is a 19-residue cyclic peptide neurotransmitter released from neurons originating primarily in the lateral hypothalamus (LH) and zona incerta (ZI) (Diniz and Bittencourt, 2017). It is produced in mammals from the cleavage of a 165-residue preprohormone (preproMCH), which also produces the MCH co-transmitters neuropeptide glutamine (E)-isoleusine-(I) and neuropeptide glycine (G)-glutamic acid-(E), and is encoded by the pro-MCH gene (*Pmch).* MCH-releasing axons are widely distributed throughout the brain, reflecting the diverse range of physiological functions and behaviors influenced by this neurotransmitter system (Diniz and Bittencourt, 2017). Similar to other hypothalamic neuropeptide systems, the MCH system is an integrator of basic homeostatic functions and of motivated behaviors, including sleep. The brain regions which receive dense innervation from MCH-releasing fibers include: the nucleus accumbens, amygdala, medial septum, and hippocampus, among others. In addition, many brain regions involved in regulating sleep and arousal are included in the long list of MCH efferent targets (e.g., the dorsal raphe, locus coeruleus, reticular formation) (Bittencourt et al., 1992). MCH neurons are mostly glutamatergic, co-releasing glutamate and MCH, along with a number of other potential co-transmitters (e.g., neuropeptide glutamine (E)-isoleusine-(I), cocaine- and amphetamine- regulated transcript, nesfatin); some MCH neurons also express markers associated with GABAergic function (Mickelsen et al., 2017). In humans, there are 2 known receptors which bind the MCH peptide, MCHR1, and MCHR2, though only MCHR1 has been well-characterized in terms of its role in behavior, due to the absence of MCHR2 expression in the most common experimental organisms (i.e., rodents). Both MCHR1 and MCHR2 are 7-transmembrane domain G-protein coupled receptors (GPCRs), but they are dissimilar in terms of their primary structures—sharing only around 40% of their amino acid sequences (Sailer et al., 2001). MCHR1 is widely distributed in the CNS, and expression is generally densest in regions that receive extensive innervation from MCH-releasing axons. In humans and primates, the distribution of MCHR2 partially overlaps with that of MCHR1, but is somewhat more restricted, and it is unclear if the two receptors are co-expressed in any individual neurons (Sailer et al., 2001; Presse et al., 2014). Because MCHR1 mediates all functions of MCH in rodents, and is the only MCH receptor which has been studied in terms of its role in sleep, it will necessarily be the main focus of this review, however, it is possible that some of these functions involve MCHR2 in humans. Activation of MCHR1 by MCH peptide is likely to have an inhibitory effect on neurons, as it is most commonly coupled to intracellular signaling through Gαi and Gαo protein subunits, which act to inhibit adenylyl cyclase and suppress production of cyclic adenosine-monophosphate (cAMP), and thereby suppress the activity of downstream effectors such as protein kinase A (PKA) and depress neuronal excitability (Presse et al., 2014). However, MCHR1 may also couple through either Gαo or Gαq/11 to activate mitogen-activated protein kinases (MAPK) including extracellular-signal related protein kinases 1 and 2 (ERK1/2) through the Ras/Raf pathway, and/or phospholipase C/protein kinase C (PLC/PKC, via Gαq), stimulating production of inositol trisphosphate (IP_3_) and increasing intracellular calcium concentrations, thereby contributing to neuronal excitability (Presse et al., 2014). Activity in the MCH system may therefore have either an inhibitory or excitatory effect on downstream regions of the CNS, depending both on the intracellular coupling of receptors in the postsynaptic neurons, and on the presynaptic release of glutamate/GABA and/or MCH and its various co-transmitters. In specific brain regions such as the shell of the nucleus accumbens (NacSh), complex synergistic interactions between intracellular signaling cascades have been reported when MCHR1 is coactivated with other GPCRs (e.g., dopamine D1 and D2 receptors) in the same neuron, possibly mediated by Gβγ subunit activity (Chung et al., 2009; Hopf et al., 2013). This complexity, potentially in combination with regionally specific innervation of the CNS by subsets of MCH neurons, may help to explain the multiple discrete functions of the MCH system, however, this has yet to be conclusively demonstrated.

In this review, we will outline the known role of the MCH system in regulating sleep behavior, and evaluate the potential clinical application of therapeutics which target the MCH system in sleep disorders. We will also review the established role of the MCH system in some non-sleep-related behaviors, which may complicate its use clinically, and discuss the recent history and current state of clinical therapies based on the MCH system.

## The role of the melanin-concentrating hormone system in sleep

**FIGURE 1:**
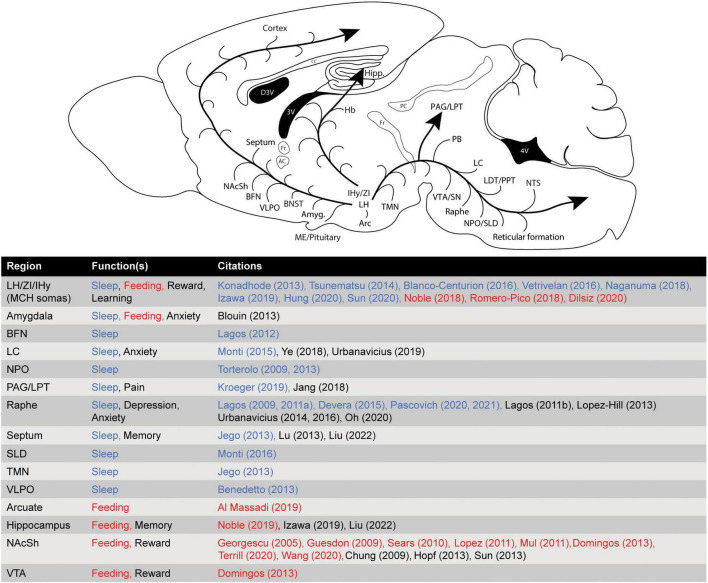
Major brain regions receiving significant innervation from the MCH system, and their corresponding behavioral function(s).

Over the past two decades, a multitude of studies have provided evidence that the MCH system has a key role in regulating arousal state (Bandaru et al., 2020). In addition to sending dense projections to brain areas implicated in the regulation of sleep and arousal, MCH neurons show increased activity during sleep. Some of the earliest evidence of the MCH system’s involvement in sleep involved looking at Fos expression, a histochemical marker of neuronal activation, during REM sleep. Rats deprived of REM sleep and allowed to recover (thus eliciting a rebound in REM sleep amount) showed increased Fos expression in MCH neurons when compared to controls (Verret et al., 2003). Another study in rats showed similar results, with MCH neurons exhibiting an increase in Fos expression after recovery from total sleep deprivation (Modirrousta et al., 2005). Single-neuron *in vivo* recording studies, using juxtacellular labeling to identify MCH neurons, also show MCH neurons to be active during sleep, with sparse activity during non-REM and maximal activity during REM sleep (Hassani et al., 2009). More recently, several studies have made use of transgenic mouse models and calcium imaging techniques to characterize MCH neurons’ activity across behaviors (see Figure 1). Izawa et al. (2019) used fiber photometry to characterize population level calcium-dependent activity in MCH neurons, showing that MCH neurons were maximally active in REM sleep and pre-REM sleep (the transition between non-REM and REM sleep), with less activity during waking and very little activity during non-REM sleep. Further characterization of single-neuron activity by several groups has shown that neurons were largely either REM- or wake-active, with a subset of neurons showing activity in both states (Blanco-Centurion et al., 2019; Izawa et al., 2019; Sun and Liu, 2020). These studies are consistent in their finding that MCH neurons are maximally active during REM sleep, with evidence for significant activity during waking, and some evidence for activity during non-REM sleep. There is also some direct evidence for the release of MCH peptide during sleep; researchers used microdialysis to sample MCH peptide tone in the amygdala of human patients (Blouin et al., 2013). They showed that MCH release was higher during sleep than during most waking experiences, but did not differentiate between sleep states. Together, these data suggest a potential functional role for MCH in the regulation of sleep states, and point to a particular association between the MCH system and REM sleep.

The main phenomenological features of REM sleep are near-complete atonia of voluntary muscles, coinciding with desynchronized electroencephalographic activity. REM sleep is also associated with dreaming. In healthy individuals, REM sleep states are always preceded by non-REM sleep, and occur periodically across sleep cycles during a night’s sleep. Like non-REM sleep, REM sleep is thought to play a role in the consolidation of memory and learning. REM sleep may be particularly important for the consolidation of hippocampal-dependent emotional memories, and for forgetting or off-loading of previously consolidated memories, through the selective strengthening or weaking of specific synapses (Hutchison and Rathore, 2015). One aforementioned rodent study has implicated REM-active MCH neurons in the active forgetting of hippocampal-dependent memories (Izawa et al., 2019); whereas wake-active MCH neurons more likely promote memory formation and enhance learning, possibly through interaction with the limbic system (Sherwood et al., 2012, 2015; Lu et al., 2013; Liu et al., 2022).

Gain- and loss- of-function studies largely support a role for MCH neurons in the regulation of REM sleep. Several studies have made use of optogenetics to activate MCH neurons in rats or mice while measuring sleep states. Optogenetic activation of MCH neurons has been shown to prolong REM sleep duration when stimulation starts at the onset of the REM state, and, in a different study, to increase non-REM to REM sleep transitions (Jego et al., 2013; Tsunematsu et al., 2014). When stimulation was more sustained, several studies have shown increased time in REM sleep, though some show an effect only during the dark period, while others show an increase during both dark and light periods (Konadhode et al., 2013; Blanco-Centurion et al., 2016). Chemogenetic activation studies, which may be of greater physiological relevance than optogenetic activation, also support the notion of a REM-promoting effect of the MCH system (Vetrivelan et al., 2016). In one study using chemogenetic activation in transgenic mice which lacked glutamatergic signaling in MCH neurons, there was still an increase in REM sleep, suggesting that MCH neurons promote REM sleep in a glutamate-independent manner, likely via MCH peptide release (Naganuma et al., 2019). Effects on non-REM sleep are less clear; stimulation during non-REM did not prolong the non-REM sleep episode (Jego et al., 2013), and contradictory effects are seen when stimulating more continuously—either an increase in non-REM sleep (in both rats and mice) (Konadhode et al., 2013; Blanco-Centurion et al., 2016), or a decrease in non-REM sleep in favor of increased REM sleep (Varin et al., 2018). Notably, both studies that demonstrated a clear increase in non-REM used AAVs to induce *Pmch*-promoter-driven expression of excitatory opsins, possibly suggesting that off-target expression underlies the observed changes in non-REM sleep, though future studies should probe this in more detail.

**FIGURE 2:**
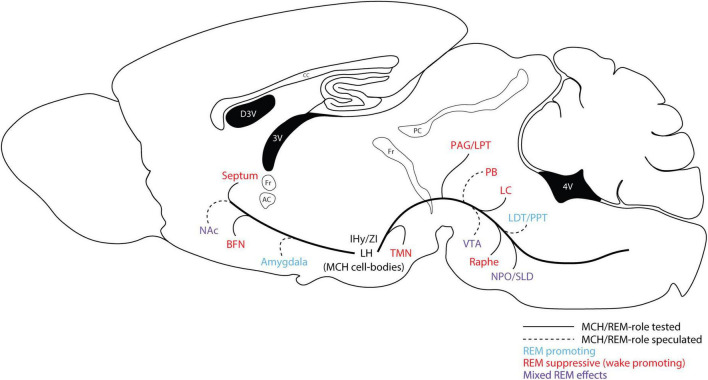
Putative brain regions participating in the REM sleep-promoting effects of the MCH system. Various REM-promoting and REM-suppressing neuronal populations/brain regions receive innervation from the MCH system. Studies have demonstrated REM-promoting effects of the MCH system through local actions in many of these regions. Generally, MCH is thought to directly inhibit REM-suppressing neurons through MCHR1/Gi/o. Multiple microinjection and optogenetic studies have demonstrated REM-promoting effects of MCH in regions such as the raphe, LC, TMN, and BFN (see Figure 1 for references). MCH may also directly or indirectly modulate activity within REM-promoting regions such as the SLD/NPO, though the evidence for this is less clear. Other brain regions are likely implicated in the regulation of specific aspects of REM sleep phenomena, or related phenomena such as cataplexy—though they are not sufficient for the generation of REM sleep—and these may also be modulated by MCH. For example, MCH projections to the VTA, the nucleus accumbens (NAc), and/or the amygdala, may modulate dopamine (DA) release (Mul et al., 2011, Domingos et al., 2013), which is likely to have downstream effects on wakefulness/sleep (Qu et al., 2008, 2010; Oishi et al., 2017), and also could have a role in cataplexy (Hasegawa et al., 2017, 2022).

Loss-of-function studies have also produced variable results. Two separate optogenetics studies demonstrated no effect on sleep architecture, though one did show a change in spectral power during REM sleep (Jego et al., 2013; Tsunematsu et al., 2014). Komagata et al. (2019) used changes in ambient temperature to modulate REM sleep propensity, and showed that MCH activation increased REM sleep even more than normal during periods of increased temperature, while inactivation of MCH neurons abolished the normal increase in REM sleep associated with a rise in temperature. To establish the circuitry through which MCH may exert its effects on REM sleep, one study used projection-specific optogenetic inhibition of MCH terminals in the ventrolateral periaqueductal gray (vlPAG) region, which reduced REM sleep when applied alone, and blocked the increase in REM sleep when combined with chemogenetic activation of the MCH system (Kroeger et al., 2019)—though multiple other brain regions or circuits have also been implicated (see Figures 1, 2 and below). These opto- and chemo-genetic studies demonstrate that manipulations of MCH neurons are capable of modulating sleep behavior in rodents, and reinforce the notion that the primary physiological role of the MCH system with regard to the sleep regulation is to promote REM sleep.

As mentioned above, MCH neurons release numerous transmitters that do not act via MCH receptors, making interpretation of studies that manipulated MCH neurons (as opposed to MCH peptide or receptors) difficult to interpret in the context of the MCH system as a clinical target. Several studies have made use of direct injections of MCH peptide and/or MCH receptor agonists/antagonists, and they too provide strong evidence for a role in sleep regulation. Intracerebroventricular (ICV) injection of MCH peptide in rats resulted in increased time in both non-REM and REM sleep (Verret et al., 2003). More targeted injections into the locus coeruleus (LC) or raphe nuclei led to increases in REM sleep (Lagos et al., 2009, 2011a; Monti et al., 2015; Devera et al., 2015, 2021; Pascovich et al., 2020, 2021), likely through actions on noradrenergic and serotonergic neurons, respectively (see below). Microinjection of MCH into the VLPO, a region implicated in generating NREM sleep, selectively enhanced NREM sleep without affecting REM sleep – however, the physiological relevance of this finding is questionable, given the lack of effect on NREM sleep observed in most subsequent studies of the MCH system (Benedetto et al., 2013). Systemic administration of MCH receptor antagonists while recording sleep state in rodents is rarer in the literature. A study using two different compounds given subcutaneously to rats showed that MCHR1 antagonism is capable of decreasing total sleep time, both non-REM and REM sleep, and increasing latency to enter sleep (Ahnaou et al., 2008). Another study, however, showed that an MCH receptor antagonist given orally did not affect sleep-wake architecture (Able et al., 2009). Transgenic mice lacking MCHR1 have subtle changes in sleep-wake architecture, paradoxically demonstrating increased REM sleep (Adamantidis et al., 2008). This result is not in agreement with other manipulations of the MCH system, but the sleep effects may be secondary to changes in other MCH-mediated behaviors and/or a result of compensatory developmental changes from the gene knockout.

The exact cellular and circuit-level mechanisms underlying the homeostatic regulation and maintenance of REM sleep, and/or the promotion of REM sleep by the MCH system, have yet to be fully elucidated. However, several studies which we highlight in this section point to certain brain regions and neuronal populations that are likely involved. Broadly, these neuronal populations can be characterized as either REM-promoting or REM-suppressing (see Figure 2)—with MCH likely acting via MCHR1 to inhibit REM-suppressing neuronal populations, and possibly to activate REM-promoting populations. The key REM-promoting and muscle atonia-generating neurons are found primarily within the brainstem. Specifically, the sublaterodorsal nucleus (SLD) within the dorsolateral pons has been identified as a major REM-promoting region, regulating both muscle atonia—through direct glutamatergic projections either to inhibitory local circuits in the spinal cord, or to descending glycinergic neurons originating in the medulla—and possibly REM sleep itself (Lu et al., 2006; Luppi et al., 2013a). It is conceivable that MCH may act directly within the SLD, either to excite glutamatergic neurons (via MCHR1/Gαq and/or MAPK pathways) or disinhibit them (via MCHR1/Gαi/o on GABAergic neurons). However, in one study where intracerebral microinjection was performed in rats, MCH infusion into the SLD was found to suppress REM sleep—ostensibly through local/direct inhibition of glutamatergic neurons (Monti et al., 2016). In the cat, the nucleus pontis oralis (NPO) is overlapping with the subcoeruleus (the anatomical equivalent of the SLD), and performs a roughly equivalent role in REM sleep as the SLD. Conversely to the rat study, microinjection of MCH directly into the NPO/subcoeruleus in the cat was found to promote REM, although the mechanism by which this occured was unclear (Torterolo et al., 2009). It is also conceivable that MCH interacts with REM-promoting cholinergic neurons within the dorsolateral pons—specifically in the pedunculopontine tegmental area (PPT) and laterodorsal tegmental area (LDT)—given the presence of MCH axons in these regions (Costa et al., 2018), however, there is no functional or behavioral data at present to support such a mechanism.

Substantially more evidence supports the idea that the MCH system promotes REM sleep through the inhibition of REM-suppressive (and/or wake-promoting) neurons found in various regions throughout the medulla, pons, and midbrain. GABAergic neurons which project from the ventrolateral periaqueductal gray area (vlPAG) and the adjacent lateral pontine tegmental area (LPT) to the SLD comprise a major REM-suppressive population. It is likely that these neurons receive inputs from, and are inhibited by the MCH system, thereby disinhibiting the SLD and promoting REM sleep, as suggested by the aforementioned study by Kroeger et al. (2019). Monoaminergic neurons (i.e., serotonergic neurons originating in the Raphe nuclei, or noradrenergic neurons originating in the LC) are generally wake-active and wakefulness-promoting, and therefore suppress REM sleep indirectly (Scammell et al., 2017). However, monoaminergic neurons may also modulate REM sleep directly through actions in the SLD or LPT (Lu et al., 2006). As previously noted, multiple studies employing region-specific microinjections of MCH peptide in the raphe or LC have demonstrated a REM-promoting effect (see above, and Figure 1). Additionally, cholinergic, glutamatergic, and GABAergic neurons originating in the basal forebrain nuclei (BFN) may be either REM-suppressing (GABA), or wake-promoting (acetylcholine/glutamate). Microinjection of MCH in the BFN was shown in one study to specifically promote REM sleep (Lagos et al., 2012; see Figures 1, 2*).*

In summary, the totality of evidence from basic science studies shows a strong functional role for the MCH system in regulating REM sleep, however, the evidence for a role in non-REM sleep regulation is much less clear, and it would be beneficial for future basic research studies to clarify this. Several sleep disorders involve dysfunctional REM sleep: in REM sleep behavior disorder, atonia promoting mechanisms are dysfunctional, leading to dream-enacting behavior which can at times be violent (Luppi et al., 2011, 2013b). Conversely, cataplexy in narcoleptics has often been characterized as an intrusion of REM sleep phenomena into waking. However, there is little research at present that supports a direct role of the MCH system in any sleep disorder, apart from a potential modulatory role in narcolepsy/cataplexy, as discussed below. Nevertheless, the growing number of studies establishing the role of MCH neurons, peptide, and receptors in sleep and arousal, underscores the potential value of MCH-based therapeutics for sleep disorders.

## The melanin-concentrating hormone system and narcolepsy

Narcolepsy is a sleep disorder that does not directly involve the MCH system, but in which targeted manipulations of the MCH system could prove an effective therapeutic approach. Narcolepsy is characterized by excessive daytime sleepiness and, in the case of type 1 narcolepsy, the sudden loss of muscle tone during wakefulness, termed cataplexy. Narcolepsy results from the loss of orexin neurons in the lateral hypothalamus, which normally promote arousal (Burgess and Scammell, 2012). These neurons are entirely non-overlapping with MCH neurons, but share many projection targets, including several that are known to regulate sleep and arousal. Orexin and MCH neurons are active at different times during waking behavior and across the sleep-wake cycle in rodents (Hassani et al., 2009; González et al., 2016). Orexin and MCH peptide release are also markedly different across behaviors in humans (Blouin et al., 2013). In addition, *ex vivo* slice physiology demonstrates that orexin can indirectly inhibit MCH neuron activity (Apergis-Schoute et al., 2015). Given that the symptoms of narcolepsy either relate to excessive sleepiness, or an intrusion of REM sleep phenomena into wakefulness—and that MCH can have a REM sleep-promoting effect—it is reasonable to ask whether the MCH system could play a role in narcolepsy symptomatology, and/or whether agents targeting MCHR1 could prove useful therapeutics for patients with narcolepsy.

Several recent studies have used animal models of narcolepsy to investigate a potential role for the MCH system. Sun and Liu (2020) used *in vivo* calcium imaging to identify the activity of MCH neurons across sleep-wake states and during cataplexy in orexin knockout mice, a murine model of narcolepsy. MCH neurons were most active during REM sleep and active wakefulness, confirming earlier studies in wild-type mice, but were least active during cataplexy. Another study showed that Fos expression in MCH neurons was positively correlated with the amount of cataplexy, but total numbers of Fos+ MCH neurons were low, despite mice being in conditions that promote cataplexy (Oishi et al., 2013). Naganuma et al. (2018) used pharmacogenetics to selectively activate MCH neurons in orexin knockout mice. With activation of MCH neurons, they observed increased cataplexy and short-latency entrances into REM sleep, consistent with the general finding that MCH promotes REM sleep phenomena. They furthermore used an MCH receptor antagonist in these mice, showing that this was able to mostly eliminate cataplexy and short-latency entrances into REM sleep. Hung et al. (2020) used a model where both orexin and MCH neurons were ablated in adult mice, demonstrating a progressive loss of both MCH and orexin neurons over a 4-week period. These mice showed decreased non-REM and REM sleep when compared to both non-lesioned mice, and orexin-only lesioned mice. The authors also observed increased cataplexy in double-ablated mice relative to orexin-ablated mice. These results are at odds with those from MCH pharmacology studies using mouse models of narcolepsy. These double-ablated mice also express other odd sleep-related behaviors, including ‘delta-theta’ sleep, a sleep state defined by high delta and theta power in the electroencephalogram, perhaps making them a less-ideal model for deriving translationally relevant insights.

While the role for the MCH system in regulating REM sleep is well established, and narcolepsy is generally thought to be a disorder of REM sleep-regulating mechanisms, the role for MCH in narcolepsy is not settled. Targeting MCH receptors may be an effective treatment for narcolepsy symptoms, particularly cataplexy, but more studies are needed.

## The role of the melanin-concentrating hormone system in other behaviors

In addition to its well-documented role in sleep regulation, the MCH system has been implicated in many other behaviors and physiological functions, including reward, learning, feeding, maternal behavior, and anxiety (Diniz and Bittencourt, 2017). As previously noted, it is likely that there are distinct subpopulations of MCH neurons that regulate some of these mutually exclusive behaviors, however, at least in rodents, all MCH peptide-dependent effects are mediated by the same receptor (MCHR1), complicating the ability to pharmacologically manipulate one behavior or function without impacting the others. Here, we summarize some of the research on these non-sleep-related functions of the MCH system, as they are likely to also be affected by MCH-targeting therapeutics.

To date, the most comprehensively characterized function of the MCH system is its role in promoting feeding. Likewise, research into MCH-based therapeutics has historically been focused on the hypothesis that interfering with this function in humans will lead to reduced food consumption and weight-loss, potentially constituting an effective treatment for obesity. Initial experiments in rodents found that exogenous MCH peptide, administered ICV, acutely increased food consumption in a dose-dependent fashion (Qu et al., 1996; Rossi et al., 1997). Despite this, chronic ICV administration of MCH was initially found to have no effect on body weight (Rossi et al., 1997). Subsequent experiments, however, established that chronic MCH treatment does indeed promote weight gain, particularly in mice provided access to palatable food (Gomori et al., 2003), for which MCH may promote a specific preference (Sakamaki et al., 2005; Morganstern et al., 2010a). In accordance with these experiments, genetic overexpression of MCH has also been found to produce hyperphagia and weight-gain in mice provided high-fat chow (Ludwig et al., 2001), while knockout of the *Pmch* gene or *MCHR1* in mice produces a lean phenotype, associated with either hypophagia (*Pmch* KO) or paradoxical hyperphagia (*MCHR1* KO), as well as hyperlocomotion and increased energy expenditure (Shimada et al., 1998; Marsh et al., 2002; Zhou et al., 2005). Though the basic research on this topic is too extensive to fully summarize here (see Al-Massadi et al., 2021; Lord et al., 2021), the emergent view suggests that MCH is not required for homeostatic feeding, but rather acts to promote non-homeostatic (hedonic) feeding, likely through its effects on reward function.

Several recent studies using optogenetic and chemogenetic techniques support the hypothesis that the MCH system enhances the reward-value of food consumption. Previously, it was shown that MCH neurons are chemosensory, increasing their firing rate in response to circulating nutrient/metabolic factors such as glucose (Kong et al., 2010). Optogenetic activation of MCH neurons, paired with a non-nutritive sweet taste (sucralose), mimics the rewarding value of sucrose, and promotes dopamine release in the striatum (Domingos et al., 2013). In another study, optogenetic activation of MCH neurons enhanced food consumption, but only if stimulation was delivered during feeding (Dilsiz et al., 2020). This study also found that activation of MCH neurons was intrinsically reinforcing, motivating operant responding even when not paired with a food-based reward. Reports on the effects of chemogenetic modulation of MCH neurons on feeding have varied. In two studies utilizing *Pmch-cre* mice, neither chemogenetic activation, nor inhibition, of MCH neurons acutely influenced food consumption (Hausen et al., 2016; Dilsiz et al., 2020). In rats, however, chemogenetic activation of a subpopulation of MCH neurons that project to the cerebral ventricles was found to promote feeding by a mechanism involving volume-transmission of MCH peptide through the CSF (Noble et al., 2018). Chemogenetic activation of MCH neurons that project to the shell of the nucleus accumbens—a brain region which is heavily implicated in reward/reinforcement, feeding, and addiction—produced acute hyperphagia in male rats and ovariectomized females, but not in intact females (Terrill et al., 2020). Another study found that MCH neuronal signaling in the hippocampus promoted impulsive food-seeking behaviors, possibly by engaging neurons that project from the hippocampus to the nucleus accumbens (Noble et al., 2019). These reports are broadly in line with earlier studies which found that local infusion of MCH peptide within the shell of the nucleus accumbens promotes feeding (Georgescu et al., 2005; Guesdon et al., 2009; Sears et al., 2010; Lopez et al., 2011; Mul et al., 2011; Sun et al., 2013; Wang et al., 2020). The MCH system is also known to promote other forms of motivated/consummatory behavior through its modulation of the mesolimbic dopamine system, including consumption of drugs of abuse (e.g., alcohol, cocaine, amphetamine) (Duncan et al., 2005; Pissios et al., 2008; Chung et al., 2009; Cippitelli et al., 2010; Morganstern et al., 2010b,^2020^; Hopf et al., 2013; Karlsson et al., 2016; see Figure 1). As previously noted, a synergistic interaction may occur when dopamine and MCH are simultaneously released onto some dopamine-responsive post-synaptic neurons within the NAcSh (D1/D2-receptor-expressing medium spiny neurons), the majority of which co-express MCHR1, possibly underlying some of the effects of MCH on motivation/reward and consumption (Chung et al., 2009; Hopf et al., 2013). It should be noted, however, that the nucleus accumbens/mesolimbic dopamine system is not the only site within the CNS where MCH acts to promote feeding. Within the hypothalamus, MCHR1-expressing proopiomelanocortin neurons of the arcuate nucleus are specifically implicated (Al-Massadi et al., 2019), as are a population of MCHR1/κ-opioid receptor-expressing neurons (Romero-Picó et al., 2018).

As with other functions of the MCH system, the relative importance of the various co-transmitters of MCH in promoting feeding is still being established; and studies employing optogenetic and chemogenetic activation of MCH neurons have generally not differentiated between the effects of MCH peptide and glutamate/GABA-mediated fast neurotransmission. The few studies which have attempted this have produced some contradictory findings. For example, although the CSF-projecting subpopulation of MCH neurons are thought to be GABAergic, and ICV infusion of GABA-agonists is known to produce hyperphagia, ICV infusion of GABA-receptor antagonists did not block the hyperphagia elicited by chemo-activation of MCH neurons—suggesting that MCH peptide exclusively mediates this effect (Noble et al., 2018). Another study, however, found that the vast majority of MCH neurons release glutamate, not GABA, during fast neurotransmission. This study also found that specifically blocking the release of glutamate from MCH neurons fully replicated the effects of MCH neuron ablation on feeding and metabolism in this study, whereas MCH peptide KO did so only partially (Schneeberger et al., 2018). This finding suggests that glutamatergic neurotransmission may be necessary for many of the MCH system’s feeding-related functions, though MCH peptide transmission alone may be sufficient for certain effects.

Some valuable insights into the role of the MCH system in feeding have been obtained through pharmacological studies with MCHR1 antagonists. These studies provide a physiologically relevant counterpoint to constitutive MCH receptor and/or peptide knockout or overexpression studies, and to injections of supra-physiological doses of MCH peptide. The most important takeaway from these studies is that MCH/MCHR1 has an ongoing physiological role in promoting hedonic feeding and setting metabolic rate. Both acute and chronic pharmacological blockade of MCHR1 reduce spontaneous food intake, or food intake stimulated by fasting or exogenous MCH (Macneil, 2013). Chronic MCHR1 blockade also produces modest reductions in body weight. In preclinical studies involving rodent models of diet-induced obesity, chronic treatment with MCHR1 antagonists resulted in reductions in body weight most commonly in the single or low-double digit percentages. Some studies have measured the effect of MCHR1 antagonists on energy expenditure and various other metabolic parameters, finding evidence that reductions in body weight result from a combination of reduced food intake and increased energy expenditure. Other metabolic parameters affected by treatment with MCHR1 antagonists include: levels of circulating glucose, insulin, leptin, and fatty acids/triglycerides, in addition to adiposity and respiratory rate (Huang et al., 2005; Mashiko et al., 2005; Kowalski et al., 2006; Gomori et al., 2007; Ito et al., 2010; Motani et al., 2013; Zhang et al., 2014; Ploj et al., 2016). Overall, these studies demonstrate the importance of the MCH system in feeding and metabolism, and the potential for MCHR1 antagonists to induce hypophagia and weight-loss.

Another function of the MCH system which may hold clinical significance is its modulatory influence on stress, anxiety and depression. The existence of this function was initially inferred based on the observation of conspicuous anatomical connectivity between MCH neurons and brain regions involved in the control of mood, anxiety, and reward (i.e., brainstem monoaminergic nuclei, the nucleus accumbens, amygdala, hippocampus). Indeed, ICV infusion of MCH peptide, or local infusion into mood-regulating brain structures, are widely reported to induce/enhance anxiety and depression-like behaviors in rodents (Georgescu et al., 2005; Lagos et al., 2011b; López Hill et al., 2013; Urbanavicius et al., 2014, 2016, 2019; Ye et al., 2018; see Figure 1), though a few early (Monzón and De Barioglio, 1999; Monzón et al., 2001), and one more recent (Oh et al., 2020), studies reported an anxiolytic or antidepressant effect. In accordance with an anxiogenic/depressive effect of MCH, systemic or intracerebral administration of MCHR1 antagonists produces anxiolytic and antidepressant-like effects in a variety of rodent behavioral assays (Borowsky et al., 2002; Chaki et al., 2005a,b, 2015; Georgescu et al., 2005; Smith et al., 2006, 2009; David et al., 2007; Millan et al., 2008; Gehlert et al., 2009; Lee et al., 2011; Ye et al., 2018). Most of these studies did not investigate the mechanism of action of MCH/MCHR1 antagonists in detail, though it may involve suppression of MCH-induced corticosterone/cortisol release. Several contradictory early reports noted either a stimulatory or inhibitory effect of exogenous MCH peptide on circulating stress hormones such as adrenocorticotropic hormone and corticosterone *in vivo* (Navarra et al., 1990; Jezová et al., 1992; Bluet-Pajot et al., 1995; Kennedy et al., 2003; Smith et al., 2006, 2009), but a stimulatory effect is best supported by the evidence in mammals. Other neurotransmitter systems that have been implicated in this mechanism include the monoamines (Georgescu et al., 2005; Marsteller et al., 2009; Ye et al., 2018), acetylcholine (Smith et al., 2006; Millan et al., 2008), and oxytocin (Phan et al., 2020).

While these studies strongly indicate a role for the MCH system in modulating stress, anxiety and depression—and suggest a potential clinical application for MCHR1 antagonists in the treatment of mood disorders—a 2006 study by Basso et al. (2006) failed to replicate many of the previously reported effects of certain MCHR1 antagonists in several of the same behavioral assays. This study also raised the issue that some reports of anxiolytic activity which were originally attributed to pharmacological MCHR1 blockade may, in fact, have been mediated by off-target receptor interactions—specifically by 5HT1A receptor agonism, similar to the mechanism of the U.S. Food and Drug Administration (FDA)-approved anxiolytic buspirone. Subsequently, a number of novel MCHR1 antagonist studies have been published which tend to reinforce the earlier findings of anxiolysis/antidepressant activity of MCHR1 antagonists (e.g., Gehlert et al., 2009; Lee et al., 2011; Chaki et al., 2015; Ploj et al., 2016; Ye et al., 2018). The previously mentioned study by Oh et al. (2020) also challenged the prevailing consensus regarding MCH’s anxiogenic/depressive effect on mood. This study demonstrated that the effects of exogenous MCH in rodent behavioral assays meant to measure anxiety or “depression” may vary, depending on the route of administration. Specifically, they reported that chronic intranasally-administered MCH peptide produced a dose-dependent anxiolytic and antidepressant-like effect in many rodent behavioral assays, and in two different preclinical chronic-stress models. Here, a mechanism of action was identified, involving intracellular signaling through mTOR, Akt, ERK, and p70S6 kinase, and synaptic plasticity in forebrain regions—similar to downstream mechanisms of known antidepressants. The divergent behavioral effects of intranasal MCH, compared with those of ICV MCH, likely result from preferential tissue-distribution within forebrain structures, as opposed to mid-/hindbrain structures. Altogether, these reports demonstrate that the role of the MCH system in mood regulation is likely more complex than what was initially assumed, and that additional, carefully replicated preclinical studies will be required before any MCH-based therapies for anxiety or depression can be considered for use in humans.

Besides being a key regulator of feeding behavior and energy metabolism—and affecting reward, addiction, stress-responses, anxiety and depression—the MCH system is also known to influence learning and memory (as previously noted) (Adamantidis and de Lecea, 2009; Sherwood et al., 2012; Izawa et al., 2019), social and maternal behaviors (Alachkar et al., 2016; Phan et al., 2020; Sanathara et al., 2021), and nociception/pain (Jang et al., 2018). It has also recently been recognized that MCH is an important regulator of ciliary growth and function. MCHR1 is widely expressed on both neuronal primary cilia, which may function like “antennae” during neurotransmission (Alhassen et al., 2022), and ependymal cilia within the walls of the cerebral ventricles. By enhancing ciliary beating, ventricularly transmitted MCH may augment volume transmission of a variety of chemical signals and metabolic factors within the CNS (Conductier et al., 2013), which may be of physiological and clinical relevance. In terms of its physiological effects, the MCH system modulates activity in the autonomic nervous system (both parasympathetic and sympathetic branches), and in the hypothalamic/pituitary (endocrine) system, exerting a downstream influence on various peripheral functions, tissues, and organs. MCHR1 is also expressed to a limited extent in the periphery, and some physiological effects of MCH/MCHR1 antagonists may be truly peripherally-mediated. Some relevant peripheral tissues/sites where MCHR1 is expressed include: adipocytes, monocytes, pancreatic β-cells, and melanocytes (Pissios et al., 2006; Al-Massadi et al., 2021). As previously alluded to, this wide-ranging functionality and distribution of the MCH system/MCHR1 may complicate the use of drugs which target it, by giving rise to side-effects.

It should also be noted that the various behavioral and physiological functions of the MCH system are not carried out in hermetic isolation from one another. Indeed, there are well-established interactions between metabolism, depression, anxiety, and sleep behavior. For instance, sleep is accompanied by a host of metabolic changes, and chronically disordered sleep is known to contribute to hormonal/metabolic dysfunction, potentially leading to obesity or diabetes. A similar relationship exists between sleep and depression, and with REM sleep in particular (Torterolo et al., 2015). Depressed patients frequently sleep to excess, exhibit reduced REM sleep-latency, and spend an increased amount of time in REM sleep. Furthermore, patients treated with antidepressant medications frequently experience little-to-no REM sleep, and REM sleep deprivation may be therapeutic in depression. Another hallmark feature of depression is a reduced capacity to experience reward, with concomitant changes in motivated behaviors. Appetite may alternately be suppressed or enhanced in depression, in a manner predicted by aberrant functional responses in the CNS; enhancement of appetite in depression is correlated with hyperfunction of the mesocorticolimbic reward circuitry, while suppression correlates with hypofunction of interoceptive regions (i.e., insula) (Simmons et al., 2016). All these interactions are potentially modulated by the MCH system, further illustrating its importance as a central integrative node in the regulation of homeostatic/motivated behaviors, and complicating its clinical/therapeutic utility. While this is not an exhaustive review of the accumulated evidence for MCH-dependent regulation of non-sleep related behaviors, it underscores the difficulty in targeting the MCH system for the treatment of sleep disorders without dysregulating other important aspects of behavior and physiology.

## Melanin-concentrating hormone drug development and application

The MCH system has historically been considered an attractive target by pharmaceutical companies, primarily due to its powerful modulatory influence over appetitive/consummatory behavior and energy metabolism. Nearly all of the rather substantial effort devoted to MCH-based drug development has been directed toward producing MCHR1 antagonists with a clinical application in obesity, though several peptide MCHR1 agonists have also been identified. The first MCHR1 antagonists were also peptidic, and these were utilized in a number of pioneering basic science studies (see MacNeil and Bednarek, 2009). At least one clinically-oriented drug development program has focused on pseudopeptide antagonists of MCHR1 (Servier, see Boutin et al., 2021); however, peptides are generally susceptible to proteolytic degradation and have limited ability to cross cellular membranes/blood-brain barrier (BBB), and are therefore considered inferior to small-molecules for most clinical applications. The first small-molecule MCHR1 antagonist was reported in 2001 (from Haynes et al., 2001). Subsequently, two papers were published in 2002, from pharmaceutical research teams at Synaptic/Lundbeck (Borowsky et al., 2002) and Takeda (Takekawa et al., 2002), which described the effects of novel small-molecule MCHR1 antagonists (SNAP-9471 and T-226926, respectively) on feeding in rodents. Following these reports, more than 20 companies commenced MCHR1 antagonist-based drug development programs over the next decade. Hundreds of patents relating to MCHR1 antagonists have been filed since the first in 1999; with annual MCHR1-related patent filings reaching their peak in 2005, a year in which 42 patents were filed (Szalai et al., 2014). However, patent-filings and commercial interest in MCHR1 antagonists significantly declined after 2012, after it was announced that an antagonist under development by Bristol-Myers-Squibb (BMS-830216) had failed its phase 1B proof-of-confidence study. To date, despite large expenditures of time and resources, and the creation of hundreds of novel MCHR1 ligands, few MCH-based drugs have advanced to clinical trials (Johansson and Löfberg, 2015). Furthermore, many of the pharmaceutical companies which operated, or continue to operate, research programs devoted to MCHR1 antagonists, have yet to advance a clinical candidate.

The first MCHR1 antagonists to enter phase 1 human safety and tolerability trials were AMG-076 (Amgen) and GW-856464 (GlaxoSmithKline); trials for both compounds commenced in 2004 (Macneil, 2013). There is little publicly available information regarding the AMG-076 trial, other than that it was discontinued, although two research papers were subsequently published describing its initial preclinical development and its effects in rodent and primate obesity models (Mihalic et al., 2012; Motani et al., 2013). GW-856464 is known to have failed due to low bioavailability. The phase 1 trial for NGD-4715 (Neurogen/Ligand) began in 2006, and in 2007 it was announced that the drug was safe and well-tolerated, and displayed some desirable effects on glucose and lipid metabolism. However, NGD-4715 also induced the cytochrome P450 enzyme CYP3A4, and was therefore liable to exhibit undesirable drug-drug interactions with pharmaceuticals commonly prescribed to the obese population (e.g., statins). Development of NGD-4715 was discontinued sometime after Neurogen was acquired by Ligand Pharmaceuticals in 2009, in favor of pursuing other molecules with improved absorption, distribution, metabolism, and excretion (ADME), and enzyme-induction properties (Tarrant et al., 2017). Alb-127158(a) (AMRI) was entered into phase 1 trials in 2011 to assess its safety, tolerability, and efficacy, and to measure plasma/CSF drug-exposure in healthy, overweight, and obese male subjects. Both a 14-day single-ascending dose, and a 14-day multiple-ascending dose (MAD) trial were conducted, with 12 subjects in the MAD trial. Moore et al. (2014) note that the drug was found to be safe and well-tolerated, with only mild adverse events reported—the most common of which were decreased appetite (more an intended effect of the drug), and headache. There were no neurological side effects or increased suicidality noted, though two subjects in the MAD trial reported experiencing depressed mood. Notably, with respect to the future development of sleep-related MCH drugs, 7 of the 12 subjects (1 of which was given placebo) reported either difficulty in falling asleep or restless sleep. These side effects generally align with basic and preclinical findings regarding the effects of MCHR1 antagonists on sleep and (to a lesser extent) mood (see previous section). The desired effect on motivation to eat was observed only at the highest dose and was only of modest size. Through analysis of CSF samples, it was determined that Alb-127158(a) did not attain sufficient CNS exposure to be efficacious at the expected dose. For this reason, further development was discontinued, though a potential clinical application of Alb-127158(a) in irritable bowel disease was suggested, based on its peripherally-selective distribution (Moore et al., 2014). As noted, the Bristol-Myers-Squibb candidate compound BMS-830216—a prodrug of BMS-819881, which likely suffered from poor pharmacokinetic/ADME properties—was evaluated in a phase 1B 28-day proof-of-confidence study that ended in 2011. However, while the drug was safe and well-tolerated, no reduction in body weight was observed in the treatment group, and phase 2 trials were therefore not initiated. More recently, a phase 1 trial to evaluate the safety, tolerability, and pharmacokinetics of Astra-Zeneca’s MCHR1-antagonist AZD1979 began in 2014. This trial was terminated due to the reaching of one or more study stopping criteria^1^, though it is unclear what this entailed. Notably, none of these MCHR1 antagonists ever advanced to phase 2 trials. However, it was quite recently announced (April 2022) that a phase 2 proof-of-concept trial would commence in order to evaluate the use of an MCHR1 antagonist known as RGH-076—under development by Gedeon Richter Plc.—as a treatment for hyperphagia/obesity in Prader-Willi syndrome. This will be the third clinical trial with this compound, meaning phase 1A/B trials were completed; though it is not immediately clear when these trials were conducted, or if the compound was identified by a different name and/or license-holder at that time.

This recent history would seem to justify a rather skeptical outlook toward the future clinical prospects of MCHR1 antagonists. However, any such determination should also weigh that most of the compounds that advanced to clinical trials were apparently considered safe and well-tolerated, and trials generally were discontinued due to a lack of efficacy in obesity-related measures, and/or lower-than-predicted CNS exposure or other ADME-related issues. There is no reason, in principle, to believe that efficacy will necessarily be a problem for sleep-related drug trials, or that the issues which led to the discontinuation of previous candidate drugs cannot be overcome. Furthermore, any future clinical drug development efforts will have at their disposal the substantial accumulated insights and improved methods and technologies which past research programs and trials have yielded. This includes significant progress in the elucidation of MCHR1’s structure-activity relationship and the identification of key ligand-binding residues (e.g., Tavares et al., 2006; Högberg et al., 2012). A 2015 review by Johansson and Löfberg (2015) offered the expert opinion that (at the time of publication) “the target [MCHR1] has not been conclusively evaluated,” and that these various “clinical failures have been associated with far from ideal compound properties.” This situation likely stems from several persistent issues that drug designers faced in the development of MCHR1 antagonists for obesity. These were primarily related to the fact that lead-compounds with high affinities for MCHR1 also tend to exhibit high affinity for the human ether-a-go-go related gene (hERG) potassium channel. Molecules which bind to and block this channel generally have low margins of safety and are liable to induce a life-threatening form of cardiotoxicity known as long QT syndrome. A molecular-modeling analysis confirmed that most MCHR1-binding chemotypes are prone to interaction with the hERG channel (Högberg et al., 2012). Certain structural modifications which suppress hERG binding can be made to putative MCHR1 ligands, however, Högberg et al. (2012) noted that “the design elements addressing the hERG issue have led to compounds that are either devoid of *in vivo* activity, or unsuitable for clinical development on the basis of their overall ADME and safety profiles.” This conclusion is borne out by the record of clinical failures detailed above. Nevertheless, subsequent advancements in the fields of machine-learning, computational chemistry, and high-throughput methods to screen for unwanted off-target affinities, may provide advantages to drug-developers not available in decades prior. For example, Lim et al. recently combined a custom-built deep-neural network machine-learning algorithm to screen a database of 357482 compounds for MCHR1 affinity, with machine-learning based *in silico* screening for hERG-related cardiotoxicity using DeepHIT (Ryu et al., 2020), to identify a novel MCHR1 antagonist lead-compound (KRX-104130). They subsequently used a transcriptome analysis-based method to identify a possible use for this compound in non-alcoholic steatohepatitis (NASH) (Lim et al., 2022).

In addition to issues related to hERG binding, some early MCHR1 antagonists not advanced to clinical trials were also known to have exhibited deficiencies in terms of their bioavailability, metabolic stability, BBB permeability, and receptor selectivity (see Luthin, 2007; Högberg et al., 2012). Some common off-target affinities of MCHR1 antagonists include serotonergic, adrenergic, histaminergic, muscarinic, and peptidergic (e.g., opioid, neuropeptide Y) receptors, as well as monoamine reuptake transporters. The research antagonists SNAP-94847 (Luthin, 2007) and ATC0175 (Chaki et al., 2005b) apparently exhibited some binding to the 5HT2B receptor—though it is unclear if they acted as agonists or antagonists. 5HT2B receptor agonist activity is undesirable in drugs intended for clinical use, as it may lead to life-threatening cardiac valvulopathy (as with the diet drug d-fenfluramine, which was pulled from the market in 1997 due to reports of cardiotoxicity) (Luthin, 2007). In preclinical studies, some MCHR1 antagonists (or MCHR1 KO models) have been reported to provoke a precipitous decline in blood pressure, or elevated heart-rate and/or convulsions (Astrand et al., 2004; Kym et al., 2005, 2006; Lynch et al., 2006; Luthin, 2007). While none of these complications appear to have arisen in the various phase 1 trials—likely because the candidate drugs were specifically designed to avoid them—it is conceivable that the additional constraints they placed on ligand design may have necessitated trade-offs in terms of molecular stability, ADME properties, or efficacy.

## Conclusion

Future MCH drug development efforts, whether focused on an application in sleep disorders or on any other functions of the MCH system, will likely need to contend with many of the same issues faced during the development of MCHR1 antagonists for obesity. Significant challenges may also arise from the myriad functions of MCH, as detailed in the previous section. At present, it seems there are few viable methods which would permit the targeting of a single given function of the MCH system, independent of the rest, in humans. One basic research study (described in the previous section) found differential CNS distribution and behavioral efficacy when contrasting intranasal MCH peptide with ICV infusion of MCH, perhaps providing a potential solution to *some* off-target drug effects: different routes of administration could, in theory, preferentially target specific tissues or brain regions, and therefore specific functions of MCH. Lastly, future research programs will likely also need to address, in some capacity, the current lack of understanding regarding the physiological roles of MCHR2 in humans, and any potential effects which might arise out of off-target ligand interactions with this receptor. As noted, the absence of a murine MCHR2 receptor has impeded efforts to define its physiological role, but it likely mediates some aspects of MCH’s function in humans. Furthermore, the ligand-binding domains of MCHR1 and MCHR2 are somewhat homologous, meaning many ligands will display affinity for both receptors (Luthin, 2007; Macneil, 2013). While it is difficult to predict all of the impediments that future efforts to drug the MCH system in sleep disorders will face, it is clear from the current literature that the challenges will be significant.

Some MCHR1 antagonists which were originally developed for use in obesity have since been considered for alternative clinical applications [e.g., Alb-127158(a) for irritable bowel disease]—though no such trials have yet commenced. The single current example of an MCHR1 antagonist entering into trials for a sleep-related clinical application has also recently been announced. In 2021, Harmony Biosciences reported their intention to submit an investigational new drug application for HBS-102 in narcolepsy/cataplexy, with a phase 2 clinical trial to commence upon acceptance. HBS-102 was previously under development by ConSynance Therapeutics under the name CSTI-100 for use in NASH, and a phase 1 (safety/tolerability) trial has already been completed. As previously noted, one preclinical study demonstrating the anti-cataplectic effect of an MCHR1 antagonist (SNAP-94748) in orexin KO mice was published in 2018 (Naganuma et al., 2018). Outside of MCHR1 antagonists for narcolepsy/cataplexy, any other potential clinical applications of drugs targeting the MCH system in the treatment of sleep disorders are entirely speculative. MCHR1 agonists could conceivably have some utility in conditions which feature a loss of REM sleep. For example, antidepressant drugs result in disruption of REM sleep, however, the relationship between REM sleep and depression is complex, and it is unclear if restoring REM sleep in patients would confer benefit or harm. Loss of REM sleep associated with clinical insomnia or otherwise insufficient sleep might also potentially be treated by an MCHR1 agonist, but as in depression, it is unclear what benefit this might provide.

Given the importance of sleep and costs associated with sleep disorders, there is value in continued interest in the MCH system as a target for novel treatments. However, there are other active areas of investigation for creating sleep therapeutics that may prove more useful than MCH-based interventions. For example, the other major lateral hypothalamic peptide system with an established role in sleep-wake regulation—the orexin system—may yield more specific or effective results, particularly for excessive daytime sleepiness and cataplexy. More basic research into the specifics of MCH’s role in sleep regulation, including identifying the principal brain regions involved and/or interactions with other neurotransmitters, will help to identify new approaches or applications of MCH agonists/antagonists that might increase their effectiveness and specificity.

## Author contributions

Both authors contributed to the conceptualizing, writing, and editing of the manuscript, and approved it for publication.

## Footnotes

1
https://www.clinicaltrials.gov/ct2/show/NCT02072993

## Abbreviations

3V, D3V, 4V: third, dorsal third, fourth ventricle
5HT: serotonin
AC: anterior commissure
ADME: absorption, distribution, metabolism and excretion
BFN: basal forebrain nuclei
cAMP: cyclic adenosine monophosphate
CC: corpus callosum
CNS: central nervous system
CSF: cerebrospinal fluid
D1: dopamine D1 (receptor)
D2: dopamine D2 (receptor)
ERK: extracellular signal-regulated kinase
Fr: fornix
GABA: γ -Aminobutyric acid
Hb: habenula
hERG: human ether-à-go-go-related gene
ICV: intracerebroventricular
IHy: incertohypothalamic area
IP_3_: inositol trisphosphate
KO: knockout
LC: locus coeruleus
LH: lateral hypothalamus
LDT: laterodorsal tegmental area
LPT: lateral pontine tegmentum
MAD: multiple ascending dose
MAPK: mitogen-activated protein kinase
MCH: melanin-concentrating hormone
MCHR1: melanin-concentrating hormone receptor 1
*MCHR1*: melanin-concentrating hormone receptor 1 gene
MCHR2: melanin-concentrating hormone receptor 2
ME: median eminence
mTOR: mammalian target of rapamycin
NAc: nucleus accumbens
NAcSh: shell of the nucleus accumbens
NPO: nucleus pontis oralis
OSA: obstructive sleep apnea
p70S6K: p70S6 kinase 1
PAG, vlPAG: periaqueductal gray, ventrolateral periaqueductal gray
PB: parabrachial nucleus
PC: posterior commissure
PKA: protein kinase A
PKC: protein kinase C
PLC: phospholipase C
*Pmch*: pro-melanin-concentrating hormone gene
PPT: pedunculopontine tegmental area
REM: rapid eye movement
RLS: restless legs syndrome
SLD: sublaterodorsal tegmental nucleus
SN: substantia nigra
TMN: tuberomammillary nucleus
VLPO: ventrolateral preoptic nucleus
VTA: ventral tegmental area
ZI: zona incerta.
